# Hyper-Methylated Loci Persisting from Sessile Serrated Polyps to Serrated Cancers

**DOI:** 10.3390/ijms18030535

**Published:** 2017-03-02

**Authors:** Angeline S. Andrew, John A. Baron, Lynn F. Butterly, Arief A. Suriawinata, Gregory J. Tsongalis, Christina M. Robinson, Christopher I. Amos

**Affiliations:** 1 Norris Cotton Cancer Center, Geisel School of Medicine at Dartmouth, 7936 One Medical Center Drive, Lebanon, NH 03756, USA; John.A.Baron@dartmouth.edu (J.A.B.); Lynn.F.Butterly@dartmouth.edu (L.F.B.); Arief.A.Suriawinata@dartmouth.edu (A.A.S.); Gregory.J.Tsongalis@hitchcock.org (G.J.T.); Christina.M.Robinson@dartmouth.edu (C.M.R.); Christopher.I.Amos@dartmouth.edu (C.I.A.); 2 Department of Medicine, University of North Carolina School of Medicine, Chapel Hill, NC 27599, USA

**Keywords:** serrated polyp, methylation, sessile serrated adenoma, colon cancer

## Abstract

Although serrated polyps were historically considered to pose little risk, it is now understood that progression down the serrated pathway could account for as many as 15%–35% of colorectal cancers. The sessile serrated adenoma/polyp (SSA/P) is the most prevalent pre-invasive serrated lesion. Our objective was to identify the CpG loci that are persistently hyper-methylated during serrated carcinogenesis, from the early SSA/P lesion through the later cancer phases of neoplasia development. We queried the loci hyper-methylated in serrated cancers within our right-sided SSA/Ps from the New Hampshire Colonoscopy Registry, using the Illumina Infinium Human Methylation 450 k panel to comprehensively assess the DNA methylation status. We identified CpG loci and regions consistently hyper-methylated throughout the serrated carcinogenesis spectrum, in both our SSA/P specimens and in serrated cancers. Hyper-methylated CpG loci included the known the tumor suppressor gene *RET* (*p* = 5.72 × 10^−10^), as well as loci in differentially methylated regions for *GSG1L*, *MIR4493*, *NTNG1*, *MCIDAS*, *ZNF568*, and *RERG*. The hyper-methylated loci that we identified help characterize the biology of SSA/P development, and could be useful as therapeutic targets, or for future identification of patients who may benefit from shorter surveillance intervals.

## 1. Introduction

Colorectal cancer (CRC) is the second leading cause of cancer death in the U.S. [1], but is preventable with colonoscopic screening and polypectomy [2]. Current recommendations for the interval between screening or surveillance examinations are based on the risks of cancer or adenomas after polypectomy. Serrated polyps, when previously all characterized as hyperplastic polyps, were not considered to have malignant potential. However, research in the last decade has revealed that serrated polyps are actually heterogeneous and that some of these lesions are likely colorectal cancer precursors [3], accounting for as much as 15%–35% of colorectal cancer incidence [4]. Under current histological definitions, more than 70% of serrated lesions are hyperplastic polyps (HPs), while 4%–25% are sessile serrated adenomas/polyps (SSA/Ps), which are the main pre-invasive serrated lesion. These are predominantly found in the proximal colon [4,5]. Current models depict a major pathway for progression via the serrated pathway involving extensive DNA methylation [6].

DNA methylation involves chemical modification of a cytosine base to form methyl-cytosine, a modification that occurs in adults at DNA sites known as “CpG loci”, which are cytosines (C) followed by guanines (G) [7]. DNA methylation patterns are passed to daughter cells during cell division, allowing for tissue-specific gene expression [8]. Altered methylation inhibits expression of genes that protect against colorectal carcinogenesis via aberrant methylation of CpG islands located in the promoter regions of tumor-suppressor genes (reviewed in [7,9,10]). The altered methylation patterns that are observed in colorectal tumors are also found in histologically normal-appearing tissue within the same colon [9,11,12]. Methylation-specific polymerase chain reaction to detect hypermethylation of the mismatch repair MLH1 promoter is currently used on routine formalin-fixed paraffin embedded tumor tissues as part of an efficient clinical test to help distinguish sporadic colorectal tumors from those likely to have germline events [13]. Thus, clinical workups including screening for methylated loci is highly feasible.

The objective of this study was to identify the hyper-methylated CpG loci detectable early in right-sided SSA/P that persist into the cancer phase of proximal serrated pathway lesion evolution. Thus, we performed a comparison of CpG island methylation among right-sided SSA/P, proximal serrated cancers, and normal mucosa.

## 2. Results

Characteristics of the New Hampshire Colonoscopy Registry right-sided SSA/P patients from Dartmouth-Hitchcock Medical Center that were included in the study are shown in Table 1. The characteristics of patients assessed for methylation were similar to those of right-sided SSA/P patients in the overall 2005–2008 registry population, with a slightly larger proportion of females (57%) and a mean age of 57 ± 6.9. All of the New Hampshire Colonoscopy Registry right-sided SSA/P samples utilized were KRAS wild-type and all except two of the polyps had BRAF V600E mutations (90%).

Using the Luo et al. [14] dataset, we generated a list of CpG loci with differential methylation in proximal CRC, compared to normal tissue. All of the 2390 CpG loci meeting the *p*-value and log-odds difference criteria were hyper-methylated. The loci with decreased methylation levels had more subtle differences, and were thus not pursued.

**Figure 1:**
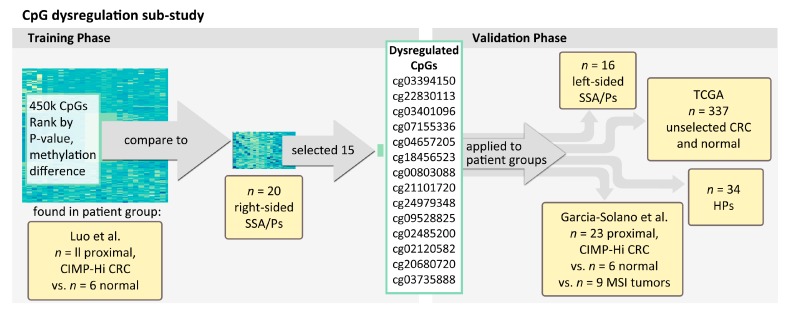
Study design diagram. The independent groups of patients assessed in the studies are depicted in yellow. The patients involved in training and validation phases of the CpG dysregulation study. CIMP, CpG Island Methylator Phenotype; CRC, colorectal cancer; TCGA, The Cancer Genome Atlas; HPs, hyperplastic polyps; MSI, microsatellite instability.

**Figure 2:**
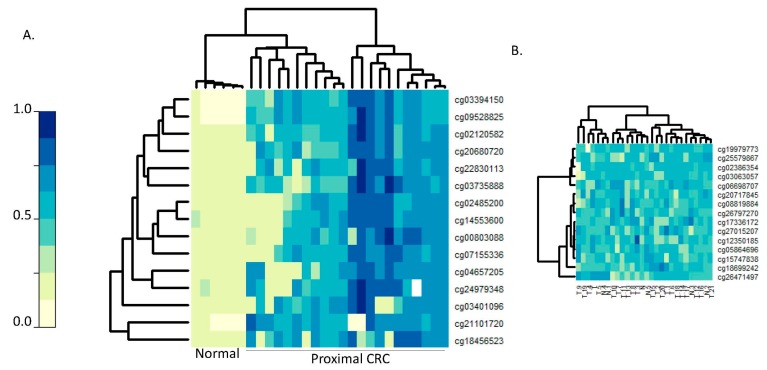
Methylation of dysregulated CpGs in proximal serrated tumors vs. normal mucosa. Proximal serrated tumors (*n* = 34) and normal mucosal samples (*n* = 15) from the study by Luo et al. [14] are depicted in columns. (**A**) Rows represent the CpGs that were dysregulated in these serrated tumors, which were restricted to the subset of 15 CpGs that were highest ranking for hyper-methylation in our right-sided SSA/Ps from the New Hampshire (NH) Colonoscopy Registry. The methylation levels are reflected by β-values from lowest (light yellow) to highest (dark blue); (**B**) The same Luo et al. [14] proximal serrated tumor and normal samples queried against a randomly selected set of 15 CpGs.

As illustrated in Figure 1, we then applied this list of CpG loci hyper-methylated in cancer from Luo et al. [14] to query the methylation levels of our New Hampshire (NH) Colonoscopy Registry right-sided SSA/Ps and selected the 15 CpG loci with the highest levels of methylation. Age and gender were not associated with the methylation status of these 15 top-ranking dysregulated CpG loci. Figure 2A shows clustering by methylation levels of the selected 15 dysregulated CpGs applied back to assess the normal vs. proximal CRC status of the Luo et al. [14] samples that were used in training. These 15 loci show strong separation between the proximal CRCs vs. normal specimens (Figure 2A). In contrast, the same Luo et al. [14] samples do not cluster by CRC status using a randomly selected set of CpG loci (Figure 2B).

We then compared the methylation levels of the 15 dysregulated CpG loci for the *n* = 20 right-sided SSA/Ps to two additional sets of polyps from our NH Colonoscopy Registry cohort: *n* = 16 left-sided SSA/P and *n* = 34 HP specimens. The methylation levels of these loci for both the left-sided SSA/Ps were similar to those of the right-sided SSA/Ps (*p* = 0.67) (Supplemental Table S1). The median methylation was lower in the HPs compared to the right-sided SSA/Ps for 12 of the 15 loci (overall HP vs. right-sided SSA/P = 0.018). The overall prevalence of the BRAF V600E mutation did not vary substantially by polyp type and anatomic location: right-sided SSA/P 90%, left-sided SSA/P 80%, right-sided HP 85%, left-sided HP 89% (χ^2^ *p* = 0.85).

**Figure 3:**
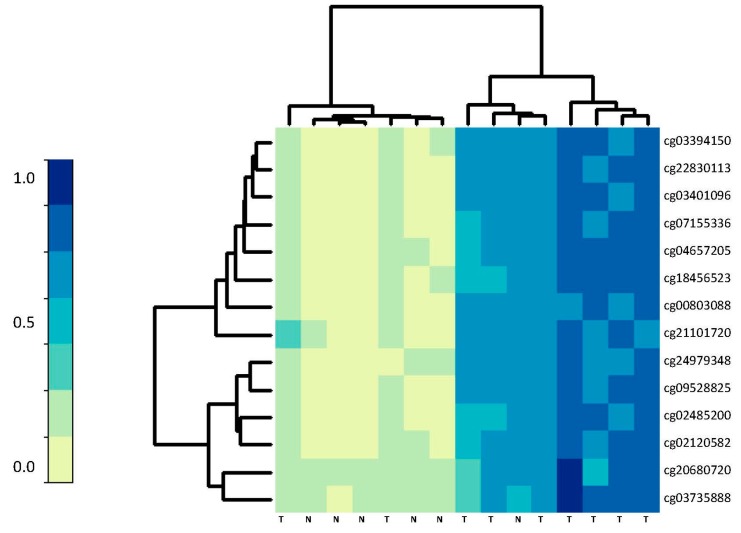
Methylation of dysregulated CpGs in MSI tumors vs. normal mucosa. An independent validation cohort of (*n* = 9) MSI tumors vs. (*n* = 6) normal mucosal samples are shown in columns. Rows represent the 15 dysregulated CpGs selected in the training datasets. The methylation levels are reflected by β-values from lowest (light yellow) to highest (dark blue). T indicates tumor, N indicates normal mucosa.

As validation of our serrated pathway markers, we then queried the methylation status of our list of 15 CpG loci in an independent population. We re-analyzed raw data from a subset of serrated CRC tumor and normal samples collected from cohorts of Spanish and Finnish patients by Garcia-Solano et al. [15]. Table 2 shows that our 15 hyper-methylated CpG loci show similar strong and statistically significant hyper-methylation in these serrated cancers (*n* = 34) compared to the normal mucosal samples (*n* = 15) (difference in log odds >0.4) collected from the patients in Spain and Finland. Likewise, we observed clustering of samples by these 15 hyper-methylated CpG loci in tumors with microsatellite instability (*n* = 9) compared to normal mucosa (*n* = 6) from the Garcia-Solano study (Figure 3).

In addition to our single probe-level analysis, we also assessed the Differentially Methylated Regions (DMRs) encompassing multiple methylation probes. We queried the 15 hyper-methylated loci within the list of DMRs identified in our serrated cancer vs. normal analysis of the Garcia-Solano et al. [15] data. The DMRs that show statistically significant hyper-methylation in serrated cancer are: *GSG1L*, *MIR4493*, *NTNG1*, *MCIDAS*, *ZNF568*, and *RERG* (Table 3).

For further validation, we assessed the methylation status of the 15 hyper-methylated CpG loci within The Cancer Genome Atlas (TCGA) dataset on colon cancer patients. These tumors were not selected on the basis of having features of any particular molecular pathway and thus represent a mixture of tumors, including those that developed via the traditional adenomatous pathway. Regression analysis showed that in comparison to the CIMP cancers, the CIMPL and non-CIMP cancers had lower levels of methylation (*p* ≤ 1.2410^−6^ for CIMP vs. non-CIMP for 13 of the 14 CpGs assayed from our hyper-methylated list of 15 CpGs, Supplemental Table S2). Tumors in the proximal colon (right-side—specifically the cecum and ascending colon) also were more common in the highly-methylated subset of tumors. The normal specimens maintained low methylation status, even though they were obtained from anatomic locations throughout proximal and distal the colon.

## 3. Discussion

We identified loci hyper-methylated in right-sided SSA/Ps that persist into serrated cancers using epigenome-wide, locus specific assessment. The major route for progression via the serrated pathway is thought to involve extensive CpG island methylation [6]. This pathway may include DNA methylation related suppression of the *MLH1* gene as a later event. The resulting loss of mismatch repair capacity leads to microsatellite instability (MSI) or further dysregulation of methylation control over cell cycle (CIMP-positive), allowing uncontrolled cell proliferation [16]. Compared to conventional carcinomas, serrated adenocarcinomas from the Garcia-Solano et al. [15] study are more likely to be CIMP high (9% vs. 30%, respectively *p* = 0.0035) [17]. Recent data show that SSA/Ps in the proximal region (right-side) of the colon are associated with a three to four-fold increased risk of concurrent advanced neoplasia and colorectal cancer [18].

Several of the hyper-methylated CpG loci in SSA/P and serrated tumors are located within genes that have been previously implicated in colorectal carcinogenesis. The hyper-methylated genes that we identified are distinct from those used to characterize the CIMP phenotype [19,20]. We observed hyper-methylation of *RET*, a tumor suppressor gene methylated in 63% of colon cancers [14,21]. Methylation of the *RET* promoter CpG island is associated with poor CRC prognosis [22]. Lower expression of the tight junction protein-encoding gene *JAM2* has been observed in colon tumors compared with normal specimens [23]. In a mouse genome-wide association study, *GSG1L* regulates glutamate receptor activity and was among the genes associated with a chromosome 15 colorectal cancer genetic susceptibility locus [24]. Immunohistochemical *BIN1* expression is low in colon cancers compared to normal tissue [25]. Thus, several of the loci hyper-methylated in SSA/P that we have identified are within genes that play important roles in established processes of carcinogenesis, but have yet to be explored mechanistically in the context of the serrated pathway to colorectal cancer.

The Differentially Methylated Regions (DMRs) identified in the analysis of serrated cancers vs. normal mucosa by Garcia-Solano et al. [15] suggest coordinated dysregulation of methylation across multiple CpG loci, defining larger regions that are particularly good candidates for clinical markers (Table 3). *NTNG1* provides axon guidance and is also a cell adhesion molecule that has been identified as a differentially methylated region in neurodevelopment [26]. *MCIDAS* is involved in the regulation of DNA replication and the mitotic cell cycle [27]. *ZNF568* is a transcriptional regulator [28]. *RERG* is a negative regulator of cell proliferation that is a close family member of the *RERGL* gene affected by a rare structural variation of 12p12.3 that increases familial colorectal cancer risk [29]. *RERG* expression is used to differentiate luminal types of breast cancer and prognostic subgroups [30].

The 15 CpG loci hyper-methylated in SSA/P also showed elevated, but more modest levels in the HPs. This suggests that these loci may be modified early in the serrated pathway. The most common subtype of HPs, the microvesicular HPs, are thought to be precursors of SSA/P due to the similarity of their histologic and molecular features [5]. More than 80% of the HPs and SSA/Ps in our registry are *BRAF* V600E mutant, which is consistent with reports in the literature [31,32]. Our observation that the methylation levels of the 15 hyper-methylated CpG loci were not distinctly different among SSA/Ps from the right vs. left sides of the bowel is likely due to our selection of a high proportion of large left-sided lesions (SSA/P ≥ 1 cm: overall 18% of right-sided, 11% of left-sided, methylation study 15% of right-sided, 24% of left-sided). Our data suggest that left-sided SSA/Ps that grow large can have similar methylation patterns to those from the right side of the bowel.

Strengths of our study included the evaluation of DNA methylation of a large number of loci from a well characterized cohort of SSA/P patients. We used several independent training and validation cohorts from different populations in order to show replicability of our findings and increase the generalizability of the results. However, limitations of this type of high-throughput analysis include assessment of a large number of loci relative to the sample size. The number of samples from the NH Colonoscopy Registry was limited, but we increased the generalizability by performing analyses across multiple studies and populations. However, the identification of large numbers of differentially methylated sites with low *p*-values suggests that our study was adequately powered for this analysis.

## 4. Patients and Methods

### 4.1. Overview

The project flow diagram in Figure 1 depicts the design of this project, which utilized methylation data run on the same platform from three separate cohorts, in addition to our own. The use of data from several cohorts was designed to increase the generalizability of our findings across populations. Details are provided below. Using data for colorectal cancer and normal mucosa from Luo et al. (GSE48684) [33], we first selected the CpG loci most hyper-methylated in the cancer tissue. These loci were assessed in our proximal SSA/Ps from the New Hampshire Colonoscopy Registry. Loci that were highly methylated in our proximal SSA/Ps were then tested in our distal SSA/Ps and HPs from the New Hampshire Colonoscopy Registry, as well as in data for serrated or colorectal cancers with high microsatellite instability (MSI-H), and normal mucosa from Garcia-Solano et al. [15]. In addition, we assessed methylation of these loci in unselected cancers and normal mucosa from The Cancer Genome Atlas (TCGA) [34].

### 4.2. New Hampshire Colonoscopy Registry

The New Hampshire Colonoscopy Registry collected data on colonoscopies performed in the state of New Hampshire [35,36], obtaining information for consenting individuals through study questionnaires at the time of colonoscopy. Endoscopists recorded detailed findings, including the anatomic location and size of polyps. Pathologic diagnosis was retrieved from pathology reports [35]. Participating patients diagnosed with serrated polyps at the Dartmouth Hitchcock Medical Center between 2005 and 2009 (*n* = 421) were eligible for this methylation study. We excluded patients with a prior history of colorectal cancer, and patients with familial adenomatous polyposis, inflammatory bowel disease, ulcerative colitis, or Crohn’s disease. Pathology slides of serrated polyps were re-reviewed by the study pathologist and classified as hyperplastic (70%), SSA/P (28%), or traditional serrated adenoma (2%). Right-sided lesions were defined as those anatomically located in the cecum, ascending colon, hepatic flexure, or transverse colon (proximal to the splenic flexure). Polyp specimens with a significant proportion of normal colorectal mucosa (>25%) and serrated polyps with dysplasia were excluded. All procedures and study materials were approved by the Committee for the Protection of Human Subjects at Dartmouth College.

### 4.3. Proximal/Right-Sided Datasets Used for Identifying Persistent Hyper-Methylated CpGs

Our objective was to identify the CpG loci dysregulated during serrated carcinogenesis that are persistently modified from the SSA/P through the CRC phases of neoplasia. We employed a two phase strategy to identify the CpGs dysregulated in both CRC and right-sided SSA/Ps. First, in the Illumina 450 k methylation data from Luo et al. (GSE48684) [33] we compared the CpG methylation levels of *n* = 23 proximal CpG Island Methylator Phenotype (CIMP) high CRCs vs. *n* = 6 normal mucosal specimens from cancer-free individuals. We ranked the CpG loci (low *p*-value, high fold-difference). Restricting to low *p*-values: ≤1.28 × 10^−7^ and a large difference in the log-odds of methylation: logFC >4 or <−4, we applied the top-ranking list of CpG loci from Luo et al. [14] to our NH Colonoscopy Registry right-sided SSA/Ps. Ranking these CpG loci by methylation level in the NH Colonoscopy Registry data, we selected the 15 loci (a priori) that were most hyper-methylated in right-sided SSA/Ps for further evaluation.

### 4.4. Datasets for Assessing Hyper-Methylated CpGs across Bowel Subsites

We compared the methylation levels of these selected 15 loci hyper-methylated in the right-sided SSA/P with six other sets of lesions. The objective of this analysis was to assess the consistency of the hyper-methylated loci in serrated lesions and cancers throughout the bowel. These lesions included:

- Left-sided SSA/P (*n* = 16) and HP specimens (*n* = 34: 55% left-sided, 45% right-sided) from the NH Colonoscopy Registry cohort.
- Colorectal cancers described in Garcia-Solano et al. GEO (GSE68060) [15] (*n* = 17 serrated adenocarcinomas vs. *n* = 11 adjacent normal mucosal samples from the Spanish cohort and *n* = 17 serrated adenocarcinomas vs. *n* = 4 adjacent normal mucosal samples from the Finnish cohort). Features used to diagnose serrated adenocarcinoma included “epithelial serrations, clear or eosinophilic cytoplasm, abundant cytoplasm, vesicular nuclei, absence of, or less than 10% necrosis of the total surface area, mucin production and cell balls and papillary rods in mucinous areas of a tumour” [17,37,38].
- Additional colorectal cancer samples that showed high microsatellite instability (MSI-H) (*n* = 9), as well as *n* = 6 normal samples from Garcia-Solano et al. [15].
- Unselected tumors from CRC patients vs. normal tissue included in the colon adenocarcinoma “COAD” dataset available from The Cancer Genome Atlas (TCGA) (*n* = 337) [34].

### 4.5. Laboratory Methods

Our NH Colonoscopy Registry studies, and those of Luo et al. [14], Garcia-Solano et al. [15], and the TCGA all utilized the same platform for assessment of DNA methylation: the Illumina Infinium Human Methylation 450 k BeadChip (for detailed methods see [15,33,34]). For the NH Colonoscopy Registry study, DNA was isolated using the QIAamp DNA Formalin-Fixed, Paraffin-Embedded Tissue Kit, according to the manufacturer’s protocol. DNA was then ligated using the REPLI-g Formalin-Fixed, Paraffin-Embedded Kit, as previously described (Qiagen Inc., Valencia, CA, USA) [39].

Epigenome-wide DNA methylation assessment was performed using the Illumina Infinium Human Methylation 450 BeadChip (Illumina, San Diego, CA, USA), which simultaneously profiles the methylation status for >485,000 CpG sites at single-nucleotide resolution, covering 96% of CpG islands, with additional coverage of island shores (<2 Kb from CpG Islands), island shelves (2–4 Kb from CpG islands), and regions flanking them. The methylation status for each CpG locus was calculated as the ratio of fluorescent signals (β = Max(M,0)/[Max(M,0) + Max(U,0) + 100]), ranging from 0 to 1, using the average probe intensity for the methylated (M) and unmethylated (U) alleles. Beta (β) = 1 indicates complete methylation; β = 0 represents no methylation. The data were assembled using BeadStudio methylation software (Illumina, San Diego, CA, USA), without normalization as per the manufacturer’s instructions.

We used the control probes included on the Illumina array to assess the quality of our samples and to evaluate potential problems such as poor bisulfite conversion or color-specific issues for each array. Methylation data were processed using the ChAMP-Chip Analysis Methylation Pipeline running in R 3.1.3 [40]. As a quality control step, probes were removed if they failed to hybridize and the median detection *p*-value was >0.01 across all samples, if they were not represented by at least three beads on the array, and samples were excluded if the fraction of failed probes associated with that sample was >0.1. All CpG loci on X and Y chromosomes were omitted from the analysis to avoid gender-specific methylation bias. The β-mixture quantile normalization method (BMIQ) was used to correct for probe design biases [41]. Technical, non-biological noise has been shown in the evaluation of samples in different batches and chips [42,43]. This problem was minimized by running all samples in one batch, mixing samples randomly across chips, and by applying the ComBat method to correct the methylation values [44]. There were *n* = 20 qualifying right-sided SSA/P samples and 387,810 CpG loci included in the analysis. We have performed pyrosequencing for technical validation of methylation levels in NH Colonoscopy Registry polyp tissues assessed using the Illumina BeadChip in our prior work [39]. Likewise, Roessler et al. [45] report the correlation between array and pyrosequencing for primary tumors (Spearman *r* = 0.86, *p* < 0.0001) [45].

### 4.6. Statistical Analysis

We identified differentially methylated loci by comparing the mean β-values of each CpG locus using empirical Bayes procedures implemented using the Bioconductor package “Limma” in the “ChAMP” analysis pipeline [40,46]. We used the “p.adjust” procedure with the Benjamini-Hochberg “BH” option to calculate *p*-values while controlling the false discovery rate (FDR). The magnitude of the differential methylation was expressed as the “logFC”, comparing the difference in the log-odds of methylation between groups (e.g., the “logFC” = 0.5 when the odds of methylation are 1.5× higher). A sensitivity analysis verified that the top ranking CpG loci were consistent when the covariates age, gender, and polyp size were included in a multivariate linear regression model using the β-value as the outcome of a generalized linear model in *R* (results not presented). Differentially methylated regions (DMRs) incorporating multiple locus positions were identified using Probe Lasso, which uses a shifting window approach based on genomic features and calculates *p*-values corrected for multiple testing with the Benjamini-Hochberg false discovery rate (FDR) method [47]. The χ^2^ test was used to assess the statistical significance of variation in the frequency of lesions by anatomic location/polyp type and mutation status. A logistic regression model was constructed using CpG methylation levels as predictors and polyp type as the outcome. We assessed the ability of this model to discriminate among polyp types by constructing a receiver operating characteristic (ROC) curve and calculating the area under that curve (AUC) (Supplemental Figure S1). Linear regression analysis was performed with the GLM procedure using tumor CIMP status as the independent variable (CIMP vs. CIMPL or non-CIMP) and the methylation level of each CpG as the outcome. We performed these statistical analyses using the *R* statistical package, version 3.13 (www.r-project.org/). Pathway Studio software, version 11.0.6.2 allowed us to assess the literature-based knowledge about the genes we identified, specifically involvement in cell processes, and relationships with other proteins (Elsevier Inc., Amsterdam, The Netherlands).

## 5. Conclusions

Refinement of serrated polyp risk assessment and optimization of the surveillance intervals for individual patients with SSA/Ps has the potential to enhance the efficacy of colorectal cancer prevention programs. Methylation assessment of CpG loci as diagnostic or prognostic biomarkers is feasible within the traditional formalin-fixed paraffin embedded clinical pipeline. Our comprehensive locus-specific assessment of DNA methylation identified loci hyper-methylated in right-sided SSA/P tissue that persists into serrated pathway tumors and is consistent across populations. The loci we identified could potentially be useful for future studies elucidating the biology of serrated pathway lesion progression, for developing therapeutic targets, or for personalizing surveillance strategies.

## Figures and Tables

**Table 1:** Characteristics of New Hampshire (NH) Colonoscopy Registry patients with right-sided sessile serrated adenomas/polyps (SSA/Ps).

Characteristics	Overall Population		Methylation Study	
	SSA/P		SSA/P	
Gender	N	%	N	%
Female	38	58	12	60
Male	27	42	8	40
Total	65	100	20	100
Age: mean, SD	56 ± 7.8		57 ± 6.9	
Polyp size				
<5 mm	26	39	11	55
5–9 mm	17	26	6	30
10–20 mm	20	30	3	15

**Table 2:** Dysregulated CpG loci queried against Garcia-Solano et al. [15] serrated tumors vs. normal.

CpG Site	Gene	Feature	Serr. Normal	Serr. Tumor	logFC	*p*-Value	BH-Corrected	Gene Name
			Mean	Mean			*p*-Value	
cg00803088	*RET*	Body-island	0.13	0.60	0.46	7.35 × 10^−12^	5.72 × 10^−10^	RET proto-oncogene
cg02120582	*PDGFD*	5′UTR-island	0.089	0.55	0.46	1.16 × 10^−11^	8.38 × 10^−10^	Platelet derived growth factor D
cg02485200	*JAM2*	1stExon-island	0.094	0.55	0.46	1.13 × 10^−9^	4.08 × 10^−8^	Junctional adhesion molecule 2
cg03394150	*GSG1L*	Body-island	0.094	0.71	0.61	1.91 × 10^−16^	1.77 × 10^−13^	GSG1-like
cg03401096	*MIR4493*	IGR-island	0.086	0.63	0.54	3.19 × 10^−13^	4.41 × 10^−11^	microRNA-4493
cg03735888	*ZNF132*	TSS200-island	0.061	0.61	0.55	4.10 × 10^−14^	8.61 × 10^−12^	Zinc finger protein 132
cg04657205	*MIR1233-1*	IGR-island	0.094	0.52	0.42	5.44 × 10^−6^	6.21 × 10^−5^	microRNA-1233-1
cg07155336	*NTNG1*	5′UTR-island	0.075	0.64	0.56	1.18 × 10^−10^	5.91 × 10^−9^	Netrin G1
cg09528825	*GSG1L*	Body-island	0.10	0.64	0.54	3.54 × 10^−17^	5.57 × 10^−14^	GSG1-like
cg14553600	*JAM2*	1stExon-island	0.12	0.53	0.41	2.27 × 10^−10^	1.03 × 10^−8^	Junctional adhesion molecule 2
cg18456523	*MCIDAS*	IGR-island	0.077	0.52	0.44	9.16 × 10^−6^	9.69 × 10^−5^	Multiciliate differentiation, DNA synthesis
cg20680720	*ZNF568*	TSS200-island	0.051	0.58	0.53	5.44 × 10^−11^	3.08 × 10^−9^	Zinc finger protein 568
cg21101720	*ANKRD13B*	Body-island	0.13	0.68	0.55	2.71 × 10^−11^	1.71 × 10^-−9^	Ankyrin repeat domain 13B
cg22830113	*BIN1*	IGR-island	0.078	0.56	0.48	1.61 × 10^−7^	2.95 × 10^−6^	Bridging integrator 1
cg24979348	*RERG*	5′UTR-island	0.091	0.52	0.43	8.02 × 10^−8^	1.61 × 10^−6^	RAS-like, estrogen-regulated, growth inhib

Benjamini-Hochberg (BH), difference in log odds of methylation (logFC).

**Table 3:** Differentially Methylated Regions (DMRs) in Garcia-Solano et al. [15] serrated tumors vs. normal.

Gene	Differentially Methylated Region				Serr. Normal	Serr. Tumor	logFC	BH-Corrected
	Start	End	Size	Core Size	Mean	Mean		DMR *p*-Value
GSG1L	28074304	28075323	1020	912	0.12	0.63	0.51	1.46 × 10^−91^
MIR4493	123228869	123229236	368	149	0.10	0.53	0.42	3.04 × 10^−26^
NTNG1	108023286	108023663	378	117	0.43	0.66	0.23	2.18 × 10^−19^
MCIDAS	54518582	54519046	465	357	0.25	0.64	0.39	1.57 × 10^−35^
ZNF568	37407207	37407307	101	69	0.10	0.59	0.49	7.35 × 10^−28^
RERG	15374496	15374673	178	50	0.07	0.42	0.35	3.43 × 10^-11^

Benjamini-Hochberg (BH), difference in log odds of methylation (logFC).

## Supplementary Materials

Supplementary materials can be found at www.mdpi.com/1422-0067/18/3/535/s1.

## Abbreviations

SSA/P	sessile serrated adenoma/polyp
