# Supplementary material for: Hyper-Methylated Loci Persisting from Sessile Serrated Polyps to Serrated Cancers
Source: Int J Mol Sci. 2017 Mar 2;18(3):535. doi: 10.3390/ijms18030535 (PMC5372551; doi:10.3390/ijms18030535)
Supplement: Supplementary file 1 [file ijms-18-00535-s001.zip › ijms-174126-suppl.pdf]

Supplemental Table 1. CpG loci are hyper-methylated in SSA/Pa on both the left and right anatomic sides of the bowel.

**Probe ID**	**Gene**	**Feature**	**SSA Left** mean	**SSA Right** mean	**logFC**	**BH-corrected P-value**
cg00803088	*RET*	Body - island	0.49	0.51	0.019	0.96
cg02120582	*PDGFD*	5'UTR - island	0.49	0.45	-0.033	0.89
cg02485200	*JAM2*	1stExon - island	0.38	0.44	0.063	0.88
cg03394150	*GSG1L*	Body - island	0.50	0.51	0.0049	0.99
cg03401096	*MIR4493*	IGR - island	0.54	0.52	-0.023	0.88
cg03735888	*ZNF132*	TSS200 - island	0.45	0.40	-0.047	0.92
cg04657205	*MIR1233-1*	IGR - island	0.38	0.47	0.087	0.88
cg07155336	*NTNG1*	5'UTR - island	0.40	0.52	0.12	0.81
cg09528825	*GSG1L*	Body - island	0.49	0.49	-0.0033	0.99
cg14553600	*JAM2*	1stExon - island	0.41	0.46	0.051	0.89
cg18456523	*MCIDAS*	IGR - island	0.49	0.56	0.076	0.88
cg20680720	*ZNF568*	TSS200 - island	0.36	0.43	0.067	0.90
cg21101720	*ANKRD13B*	Body - island	0.55	0.53	-0.021	0.97
cg22830113	*BIN1*	IGR - island	0.41	0.55	0.14	0.78
cg24979348	*RERG*	5'UTR - island	0.35	0.40	0.050	0.93

Benjamini-Hochberg (BH), difference in log odds of methylation (logFC)

Supplemental Table 2. Dysregulated CpG loci differ by CIMP staus in The Cancer Genome Atlas (TCGA) data.

		P-value	
**Probe ID**	**Gene**	**CIMPL vs. CIMP**	**non-CIMP vs. CIMP**
cg00803088	*RET*	0.0301	< 2e-16
cg02120582	*PDGFD*	0.00221	5.80E-11
cg02485200	*JAM2*	0.000672	< 2e-16
cg03394150	*GSG1L*	0.537	9.81E-09
cg03401096	*MIR4493*	0.004	1.72E-08
cg03735888	*ZNF132*	0.171	9.24E-09
cg04657205	*MIR1233-1*	1.30E-13	< 2e-16
cg07155336	*NTNG1*	1.20E-05	< 2e-16
cg09528825	*GSG1L*	0.436	6.94E-09
cg14553600	*JAM2*	0.00997	3.83E-14
cg18456523	*MCIDAS*	0.000398	1.24E-06
cg21101720	*ANKRD13B*	0.847	0.671
cg22830113	*BIN1*	9.98E-11	< 2e-16
cg24979348	*RERG*	6.23E-06	9.10E-15
