# Supplementary material for: Hyper-Methylated Loci Persisting from Sessile Serrated Polyps to Serrated Cancers
Source: Int J Mol Sci. 2017 Mar 2;18(3):535. doi: 10.3390/ijms18030535 (PMC5372551; doi:10.3390/ijms18030535)
Supplement: Supplementary file 1 [file ijms-18-00535-s001.zip › ijms-174126-figure.pdf]

Supplemental Figure 1.

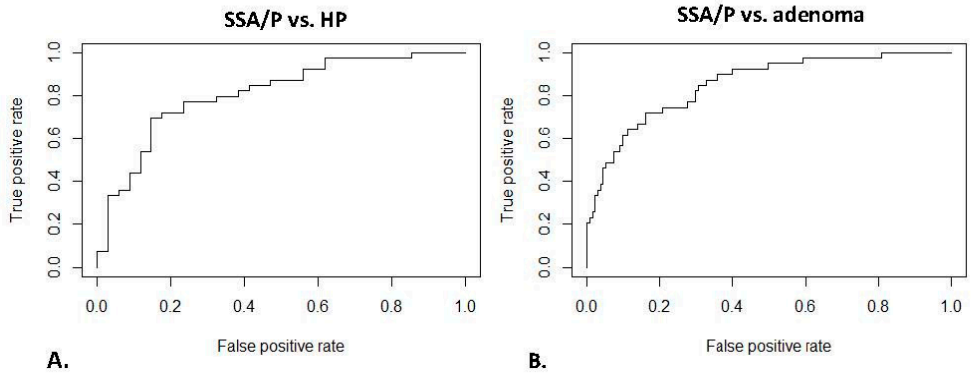

A.

B.

Supplemental Figure 1. Hyper-methylated CpGs discriminate SSA/P vs. HP or adenoma. We constructed logistic regression models using data from the NH Colonoscopy Registry containing the methylation level of the 15 hyper-methylated CpG loci as predictors and polyp type as the outcome: (**A**) SSA/P (n = 39) vs. HPs (n = 34), (**B**) SSA/P (n = 39) vs. conventional adenoma (n = 135). We assessed the ability of this model to discriminate among polyp types by plotting the true positive rate against the false positive rate at various threshold settings as a receiver operating characteristic (ROC) curve and calculating the area under that curve (AUC).
